# Prenatal Lipopolysaccharide Exposure Promotes Dyslipidemia in the Male Offspring Rats

**DOI:** 10.3389/fphys.2018.00542

**Published:** 2018-05-16

**Authors:** Shiyun Yu, Yan Wen, Jingmei Li, Haigang Zhang, Ya Liu

**Affiliations:** ^1^Department of Pharmaceutics, College of Pharmacy, Institute of Materia Medica, Third Military Medical University, Chongqing, China; ^2^Department of General Surgery, Southwest Hospital of Third Military Medical University, Chongqing, China; ^3^Department of Pharmacology, College of Pharmacy, Third Military Medical University, Chongqing, China

**Keywords:** intrauterine environment, inflammation, offspring, dyslipidemia, mitochondrion

## Abstract

Inflammation is critical to the pathogenesis of cardiovascular diseases (CVDs). We have uncovered intrauterine inflammation induced by lipopolysaccharide (LPS) increases CVDs in adult offspring rats. The present study aimed to explore the role of prenatal exposure to LPS on the lipid profiles in male offspring rats and to further assess their susceptibility to high fat diet (HFD). Maternal LPS (0.79 mg/kg) exposure produced a significant increase in serum and hepatic levels of total cholesterol, triglycerides, low-density lipoprotein cholesterol, aspartate amino transferase as well as liver morphological abnormalities in 8-week-old offspring rats. Meanwhile, disturbed gene expressions involved in hepatic lipid metabolism and related signaling pathways were found, especially the up-regulated very-low density lipoprotein receptor (VLDLR) and down-regulated transmembrane 7 superfamily member 2 (TM7SF2). Following HFD treatment, however, the lipid profile shifts and liver dysfunction were exacerbated compared to the offsprings treated with prenatal LPS exposure alone. Compared with that in control offsprings, the hepatic mitochondria (Mt) in offspring rats solely treated with HFD exhibited remarkably higher ATP level, enforced Complex IV expression and a sharp reduction of its activity, whereas the offsprings from LPS-treated dams showed the loss of ATP content, diminished membrane potential, decline in protein expression and activity of mitochondrial respiratory complex IV, increased level of MtDNA deletion as well. Furthermore, treatment with HFD deteriorated these mitochondrial disorders in the prenatally LPS-exposed offspring rats. Taken together, maternal LPS exposure reinforces dyslipidemia in response to a HFD in adult offsprings, which should be associated with mitochondrial abnormalities and disturbed gene expressions of cholesterol metabolism.

## Introduction

Cardiovascular disease (CVD) is a leading cause of death worldwide. Despite recent advances in lifestyle changes and pharmacologic treatments, dyslipidemia is indeed the highest contributor to the occurrence of CVD among all modifiable risk factors. Notably, hyperlipidemia is present in a substantial proportion of young adults, according to data from the National Health and Nutrition Examination Survey (NHANES) of USA (Pencina et al., 2014; Kit et al., 2015). Accumulating epidemiological evidence has suggested that risk trajectories that cause adult CVD may already be present in the early postnatal period or at birth, and hyperlipidemia in early adulthood can increase long-term risks of coronary heart disease (Barker, 2004; Cleal et al., 2007; Navar-Boggan et al., 2015). Thus, there is a substantial focus on the developmental origins of CVD and the early determinants of cardiovascular risk.

Inflammation is central to the pathogenesis of CVD and partially mediates the effects of the traditional risk factors (Libby, 2012; Golia et al., 2014). Conditions characterized by chronic inflammation, such as systemic lupus erythematosus, rheumatoid arthritis, and Crohn's disease have been reported to be related to accelerated CVD morbidity (Gkaliagkousi et al., 2012; Hong et al., 2015; Bacchiega et al., 2017). In addition, patients with acute coronary syndromes have elevated levels of inflammatory markers (Libby et al., 2014). It has been indicated that maternal experienced inflammation is very common during pregnancy, such as inflammation in periodontal and urinary tract, or respiratory infections and even air pollution, maternal obesity, however, they are usually ignored in the context of fetal development (Seaton et al., 1995; Ramsay et al., 2002). As a result of maternal inflammation, the fetus may be exposed to increased levels of cytokines, chemokines, and/or lipid mediators from mother through the placental circulation. Thus inflammatory events during early life may play a key role in programming for later CVDs susceptibility. Recently, we developed a well-characterized rat model of the maternal non-bacterial inflammation induced by lipopolysaccharide (LPS) or zymosan during pregnancy, which displayed elevated blood pressure as early as 6 weeks of age in offspring rats, moreover, blood pressure progressively elevated with age (Wei et al., 2007; Hao et al., 2010a). In addition, the 12-week-old offspring exhibited early pathological alterations in the aorta related to atherosclerosis (Zhao et al., 2014). Subsequently, cardiac hypertrophy and cardiac insufficiency were detected in 16 and 32-week-old offspring, respectively (Wei et al., 2013). However, the role of LPS challenge during early life on lipid profiles in adult offspring rats and its underling mechanism remain largely unclear. To date, it is well known that mitochondria are the main organelles devoted to lipid metabolism. Mitochondrial dysfunction occurs in the context of obesity, insulin resistance, and non-alcoholic steatohepatitis (NASH) (Serviddio et al., 2008; Cheng and Almeida, 2014; Fisher-Wellman et al., 2014). Importantly, mitochondria could provide an ideal candidate mechanism for adult diseases resulted from maternal stresses, because mitochondria are maternal inheritance (Dillin et al., 2002). Herein, we hypothesized mitochondrial disorders should contribute to lipid abnormalities in offspring rats from LPS-exposed dam.

In the present study, the rat model of prenatal LPS exposure was introduced to address the impact of maternal inflammation during pregnancy on blood lipid profiles in 8-week-old offspring rats, and further to assess their susceptibility to dyslipidemia and mitochondrial dysfunction in adult male offspring challenging with the well-established risk factors for CVD, including high fat diet feeding.

## Materials and methods

### Animals

Nulliparous pregnant time-mated Sprague–Dawley rats were obtained from the Experimental Animal Center of the Third Military Medical University (Chongqing, China). All animals had free access to standard laboratory rat chow and tap water. Until parturition, rats were housed individually in a room at constant temperature (24°C) and under a 12 h light–dark cycle. The pups were raised with a lactating mother until they were weaned, then they were removed to cages containing three or four other rat pups. The present study conforms to the *Guide for the Care and Use of Laboratory Animals* published by the US National Institutes of Health (NIH Publication *N*. 85–23, revised 1996; http://www.nap.edu/readingroom/books/labrats/index.html). The study protocol was approved by the Ethical Committee for Animal Experimentation of the Third Military Medical University.

After 5 days of acclimation, the pregnant rats were randomly divided into 2 groups (*n* = 8 in each), i.e., the control group and the LPS group. The rats in these two groups were administered with normal saline 0.5 ml and 0.79 mg/kg LPS (Sigma Chemical, St. Louis, MO, USA) intraperitoneally, respectively, on gestation days 8, 10, and 12, which are important time points for embryonic liver development (Si-Tayeb et al., 2010). The litters were counted and weighed after birth. The litter size was then reduced to eight pups to ensure equal nutrient access for all the offspring. Neonatal rats were cared for by their mothers until 4 weeks of age. At 5-week-old, the offspring rats from the control and LPS groups were both randomly divided again into 2 groups and were assigned to the control (3.85 kcal/g; D12450B; Research Diets, New Brunswick, NJ), or a high-fat rat chow (5.24 kcal/g; D 12492, Research Diets). The composition of the two diets is shown in Supplemental Table 1. Drinking water and food were available *ad libitum* throughout the study. At 8 weeks of age, the male offspring from each group were selected randomly for the following assays.

### Body weight and food intake

The body weight of the male offspring rats at the age of 1 day were monitored, subsequently, body weight were monitored weekly during the experiments, and food intake was measured daily manually.

### Determination of blood lipid, ALT and AST levels and hepatic lipid contents

At 8 weeks of age, the male offspring from each group were selected randomly, fasted overnight and anesthetized with sodium pentobarbital (50 mg/kg) administered intraperitoneally. Blood was collected by removal of the eyeballs and centrifuged by 3,000 × g for 15 min. Then, supernatants were collected to serve as the plasma samples for further assay. The levels of serum lipids, including total cholesterol (TC), triglycerides (TG), high-densitylipoprotein cholesterol (HDL-C), low-density lipoprotein cholesterol (LDL-C), alanine aminotransferase (ALT) and aspartate amino transferase (AST) were detected using an AU-2700 automatic biochemical analyzer (Olympus, Tokyo, Japan). Contents of TC, HDL-C, LDL-C (Abcam, No. ab65336) and TG (Abcam, No. ab65390) in liver was quantified using a colorimetric kit, respectively, according to the manufacturers' protocol.

### Histological analysis

Fixed liver sections were stained with hematoxylin and eosin (H&E) for visual observation of steatosis and cellular infiltration. Additionally, alternate sections were stained with Oil Red O for analysis of lipid accumulation. The Kleiner scoring system, a feature-based semiquantitative scoring system, was also introduced to quantify the histological changes in liver (Kleiner et al., 2005). Briefly, an activity score was generated by adding the individual scores as below: steatosis (<5% = 0, 5% - 33% = 1, 33% - 66% = 2, > 66% = 3); ballooning (none = 0, few = 1, prominent = 2); and lobular inflammation (none = 0, <2 foci = 1, 2-4 foci = 2, > 4 foci = 3). A score of less than 3 refers to mild nonalcoholic fatty liver (NAFL), a score of 3 to 4 refers to moderate NAFL, and a score of 5 or more correlates with non-alcoholic steatohepatitis (NASH). The average score for each histological features in each group was provided.

### Super array analysis

The Rat Lipoprotein Signaling and Cholesterol Metabolism RT^2^- Profiler PCR array (Qiagen, Cat. No. PARN-080Z) was used to determine the effects of maternal LPS exposure during pregnancy on the expression of 84 genes related to lipoprotein transport and cholesterol metabolism. Briefly, the total RNA was isolated from the livers of the 8-week-old offspring rats fed a normal diet from the maternal control and LPS exposure groups, and then cDNA was prepared by using a real-time RT-PCR array first strand kit. A total volume of 25 μl of the PCR mixture, which included 12.5 μl of RT^2^ Real-Time SYBR Green/ROX PCR master mix from Qiagen, 11.5 μl of double-distilled water, and 1 μl of template cDNA was loaded in each well of the PCR array. PCR cycles were performed according to the manufacturer's protocols. Data were analyzed using the comparative Ct method with normalization of the raw data to housekeeping genes including β-glucuronidase, hypoxanthine phosphoribosyl transferase 1, heat shock protein β-1, GAPDH, and β-actin. Controls including rat genomic DNA contamination, reverse transcription and positive PCR were performed simultaneously.

### RNA preparation and real-time reverse transcription-PCR

To verify the results of super array, real-time PCR analysis was performed as described previously (Hao et al., 2010b). The PCR primers used were designed by Premier 5.0 (PREMIER Biosoft International, Palo Alto, CA, USA) based on published nucleotide sequences for rat VLDLR (forward: 5′-ATG TGA CAG CTC CCA ATT CC-3′; reverse: 5′-GCT TTC ATC AGA ACC ATC TTC AC-3′), rat TM7SF2 (forward: 5′-GCC TCG GTT CCT TTG ACT TC-3′; reverse: 5′-CCA TTG ACC AGC CAC ATA GC-3), and rat β-actin (forward: 5′-ACG GTC AGG TCA TCA CTA TCG-3′; reverse: 5′-GGC ATA GAG GTC TTT ACG GAT G-3′).

### Mitochondrial membrane potential assay

Primary hepatocytes from 8-week-old offspring rats were isolated and cultured from liver tissues using a standard protocol. Cells were washed with PBS, stained with 1 μM tetramethylrhodamine-methyl ester-perchlorate (TMRM, Invitrogen, Carlsbad, CA, USA) for 20 min in PBS at 37°C, and then the cells were washed three times again. The relative fluorescence intensity was quantified immediately using the GloMax® Discover System (Promega) with 548 nm excitation and 574 nm emission, respectively, and the results were expressed as % control.

### ATP measurements

ATP content in the liver tissue was determined by using the ATP Assay kit (Abcam, Cambridge, MA). Ten milligrams of flash frozen tissue was used to perform the colorimetric assay following the manufacturer's instructions. The ATP content was measured in duplicate and expressed as % control.

### Quantitative PCR for MtDNA damage

The mtDNA 4977-bp common deletion was detected as previously described with modifications (Partridge et al., 2007). PCR products were generated using primers to identify a 235-bp control product and a 300-bp product spanning the rat equivalent of the human common 4977-bp deletion. Briefly, DNA was extracted from liver tissue samples using a DNAZol assay kit (Invitrogen, Carlsbad, CA, USA) and quantified by spectrometry. PCR products were initially generated using generic primers to identify the presence of the control and the region flanking the human equivalent of the “common” 4977-bp deletion. Products were gel-extracted, sequenced, and the qPCR primers were designed based on these sequences. Control: forward: 5′-CCT CCC ATT CAT TAT CGC CGC CCT TGC-3′, reverse: 5′-GTC TGG GTC TCC TAG TAG GTC TGG GAA-3′. Primers generated a 235 bp fragment. Mutant primers designed to incorporate the predicted break point: forward: 5′-AAA ATC CCC GCA AAC AAT GAC CAC CC-3′, reverse: 5′-GGC AAT TAA GAG TGG GAT GGA GCC AA-3′, producing a 300-bp product. Both sequences were screened by BLASTn to eliminate potential redundancies in the rat nuclear genome. They were also screened against the NCBI SNP database and the Japanese Mitochondrial SNP database. The reaction was performed in a total volume of 25 μl with QPK-201 SYBR Green PCR Master Mix (Takara, Shiga, Japan) under the following conditions: after an initial 2 min at 95°C, the samples were submitted to 40 cycles of 20 s at 94°C for denaturation and 30s at 60°C for annealing, and 45 s at 72°C for elongation. Finally, the samples were submitted to 10 min at 72°C to end this reaction. Serial dilutions of known control DNA were used for generating standard curves. All samples were amplified in triplicate. Absolute quantification was performed using the standard curve method and represented as “fold increase” over the controls.

### Western blot analysis

The samples from liver were processed and western blot analysis was performed as reported (Tong et al., 2017). Primary antibodies, including anti-Complex I (0.5μg/ml Santa Cruz Biotechnology, Inc., USA), anti-Complex II (0.4 μg/ml, Santa Cruz Biotechnology, Inc., USA), anti-Complex III (1.25 μg/ml, Abcam, Cambridge, MA, USA), anti-Complex IV (1 μg/ml, Invitrogen, Carlsbad, CA, USA), and anti-Complex V (1 μg/ml, Abcam, Cambridge, MA, USA) antibody were used.

### Enzymatic activities of complex IV

The Complex IV activity was assayed using a biochemistry kit (Sigma, St. Louis, MO, USA). Briefly, mitochondrial fractions from 8-week-old offspring rats were isolated from fresh liver tissues using a mitochondrial isolation kit (Pierce, Rockford, IL, USA). COX activity was detected by measuring the oxidation of reduced cytochrome C at 550 nm over 60 s (nmol oxidized cytochrome C per min per mg protein). The enzyme activities were calculated with the following: Units/ml = (ΔA/min × dil × 1.1)/((vol of enzyme) × 21.84).

### Statistical analysis

The results are expressed as the mean ± standard error of the mean (SEM). The data were analyzed by one-way analysis of variance (ANOVA) with Bonferroni *post-hoc* test (Graph Pad Prism version 5.01, Graph Pad Software, La Jolla, CA). The value of *P* less than 0.05 was considered to be statistically significant.

## Results

### Alterations in body weights in offspring rats

**Figure 1:**
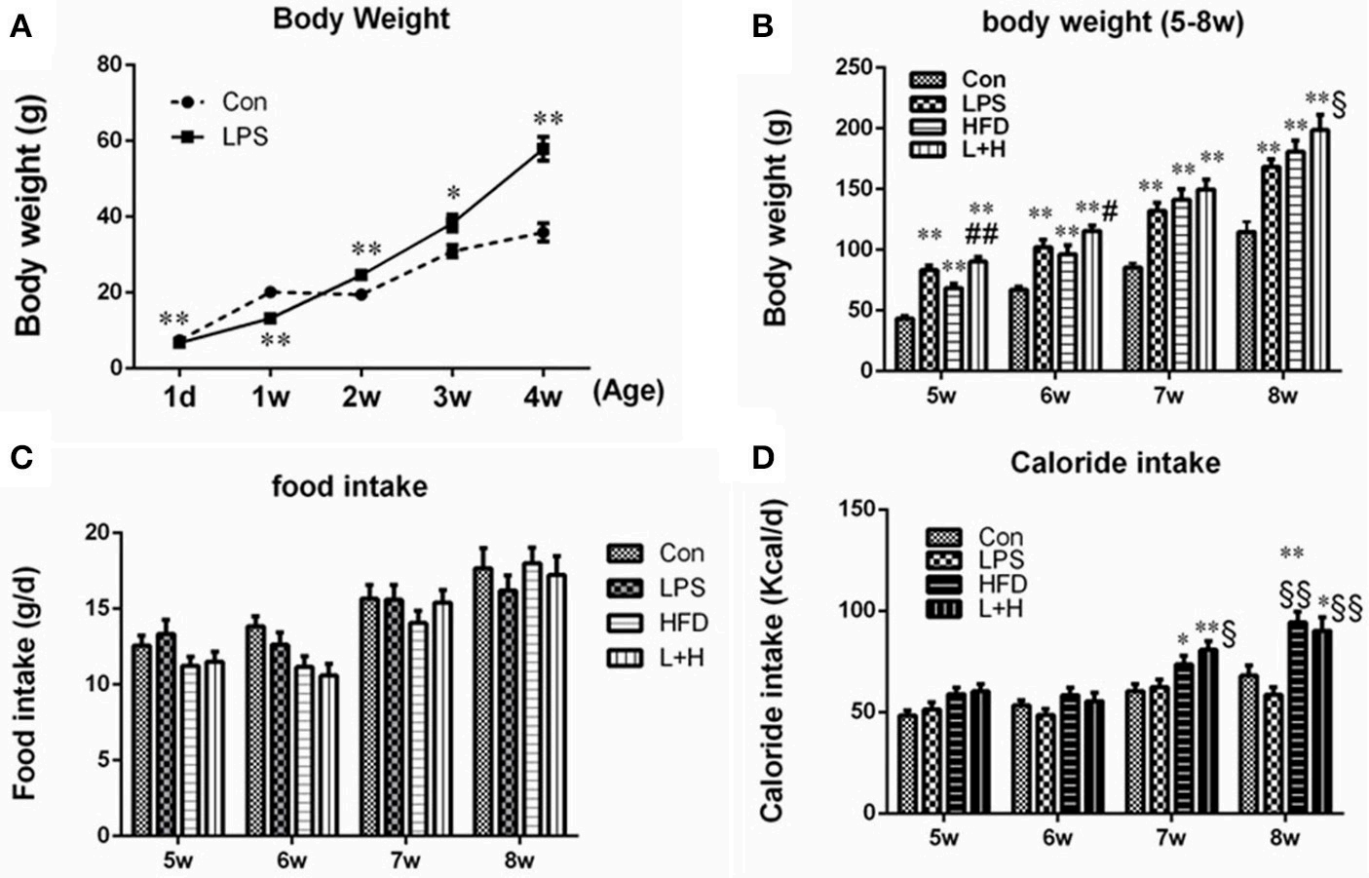
Body weights in offspring rats at the age of 1 day, 1, 2, 3, and 4 weeks **(A)**. Body weights in offspring rats from 5 to 8 weeks old **(B)**. Food intake **(C)** and caloride intake **(D)** in offspring rats from 5 to 8 weeks old. The data are presented as the mean ± SEM. *n* = 10 in each group. ^*^P < 0.05, ^**^P < 0.01 vs. control, ^§^*P* < 0.05, ^§§^*P* < 0.01 vs. LPS group, ^#^*P* < 0.05, ^##^*P* < 0.01 vs. HFD group (One-way ANOVA). Con group, male offspring rats treated with prenatal saline and postnatal normal diet from 4 to 8 weeks old; LPS group, male offspring rats treated with prenatal LPS and postnatal normal diet from 4 to 8 weeks old; HFD group, male offspring rats treated with prenatal saline and postnatal high fat diet from 4 to 8 weeks old; L+H group, male offspring rats treated with prenatal LPS and postnatal high fat diet from 4 to 8 weeks old.

Compared to the pups in the control group, the body weight in the prenatal LPS exposure group was significantly decreased at birth (6.74 ± 0.04g vs. 7.43 ± 0.05 g, *n* = 10, *P* < 0.01) and 1 week of age (*P* < 0.01). However, the pups from dams who were exposed to LPS during pregnancy consistently gained body weight from 2 weeks of age (*P* < 0.05 or 0.01) (Figure 1A). After lactation, the pups in the control and LPS groups were both divided and submitted to either a standard or a high fat diet, respectively, from 5 to 8 weeks of age. Although daily food intake of offspring rats among the four groups was not significantly different in mass, HFD and prenatal LPS exposure plus high fat feeding groups both consumed significantly increased calories at the age of 7 and 8 weeks when compared with control or prenatal LPS exposure offspring rats (*P* < 0.05 or 0.01), because high fat diet is calorically more dense than the control diet (Figures 1C,D). As shown in Figure 1B, the offsprings' body weights in the LPS, HFD and prenatal LPS exposure plus high fat feeding groups all increased dramatically (*P* < 0.01) compared with the control group from 5 to 8 weeks of age. Compared with offspring from the dams exposed to LPS, high fat diet alone caused less body weight gain in offspring at 5 weeks of age (HFD vs. LPS, *P* < 0.05), whereas combination treatment produced significantly increased body weights in 8-week-old offspring (L+H vs. LPS, *P* < 0.05). In addition, offspring with the treatment of maternal LPS exposure and high fat feeding showed significantly increased body weights at 5 (L+H vs. HFD, *P* < 0.05) and 6 (L+H vs. HFD, *P* < 0.01) weeks of age compared with the offspring treated solely with the a postnatal high fat diet.

### Serum lipid disorders in offspring rats

**Figure 2:**
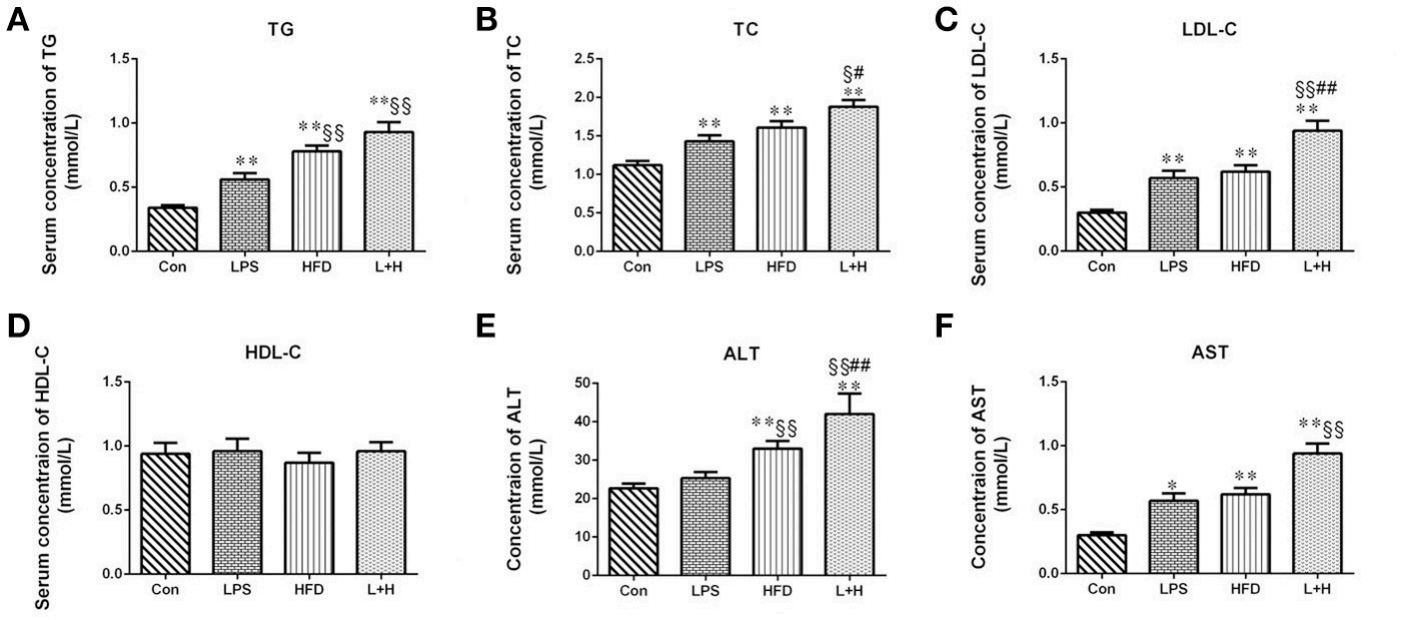
Serum levels of total cholesterol **(A)**, triglycerides **(B)**, low-density lipoprotein cholesterol **(C)**, high-density lipoprotein cholesterol **(D)**, alanine aminotransferase **(E)**, aspartate amino transferase **(F)** in 8-week-old offspring rats. The data are presented as the mean ± SEM. *n* = 6 in each group. ^*^P < 0.05, ^**^P < 0.01 vs. control, ^§^*P* < 0.05, ^§§^*P* < 0.01 vs. LPS group, ^#^*P* < 0.05, ^##^*P* < 0.01 vs. HFD group (One-way ANOVA). Con group, male offspring rats treated with prenatal saline and postnatal normal diet from 4 to 8 weeks old; LPS group, male offspring rats treated with prenatal LPS and postnatal normal diet from 4 to 8 weeks old; HFD group, male offspring rats treated with prenatal saline and postnatal high fat diet from 4 to 8 weeks old; L+P group, male offspring rats treated with prenatal LPS and postnatal high fat diet from 4 to 8 weeks old.

Obviously, any treatment, including prenatal LPS exposure, postnatal high fat feeding, and combination treatment caused elevated serum levels of TC (*P* < 0.01), TG (*P* < 0.01) and LDL-C (*P* < 0.01) in the offspring at 8 weeks of age by comparison of those in control group, respectively. In addition to significantly increased TG levels (HFD vs. LPS, *P* < 0.01) in the offspring from the dams exposed to LPS, the levels of TC and LDL-C were not markedly different between the offspring treated with prenatal LPS exposure and postnatal high fat diet feeding. On the other hand, compared to the offspring from dams exposed to LPS alone, offspring treated with prenatal LPS exposure and postnatal high fat diet presented with dramatically higher TG (L+H vs. LPS, *P* < 0.01) and LDL-C (L+H vs. LPS, *P* < 0.01) levels. Nevertheless, the offspring that received the combination treatment demonstrated the most deteriorated hyperlipidemic profiles, featured with significantly elevated levels of TC (L+H vs. HFD, *P* < 0.05) and LDL-C (L+H vs. HFD, *P* < 0.01) compared with the high fat diet fed offspring. Simultaneously, there was no significant difference in the serum HDL-C level among the offspring from different treatment groups (Figures 2A–D).

### AST and ALT levels in offspring rats

To assess the liver function, the serum activity of ALT and AST were detected in the 8-week-old offspring. It was showed that the single prenatal LPS exposure induced significantly increased level of AST in the serum (LPS vs. Con, *P* < 0.05). And the levels of ALT and AST in the offspring with the exposure to a high-fat diet alone or synergistic treatment of prenatal LPS exposure and high-fat diet feeding were both markedly elevated (HFD vs. Con, *P* < 0.01; L+H vs. Con, *P* < 0.01) relative to the level in the control offspring. Compared to the offspring in the prenatal LPS exposure group, ALT level was significantly higher (HFD vs. LPS, *P* < 0.01) in the control offspring challenged with 4 weeks of a high-fat diet. Furthermore, prenatal LPS exposure accompanying 4 weeks of high-fat diet treatment led to significantly higher serum levels of ALT and AST as compared with the prenatal LPS exposure offspring (L+H vs. LPS, *P* < 0.01). In contrast, the serum AST level in maternal LPS exposure offspring plus high-fat diet feeding was obviously increased compared with that in the offspring treated with a high-fat diet alone (L+H vs. HFD, *P* < 0.01) (Figures 2E,F).

### Hepatic lipid profiles in offspring rats

**Figure 3:**
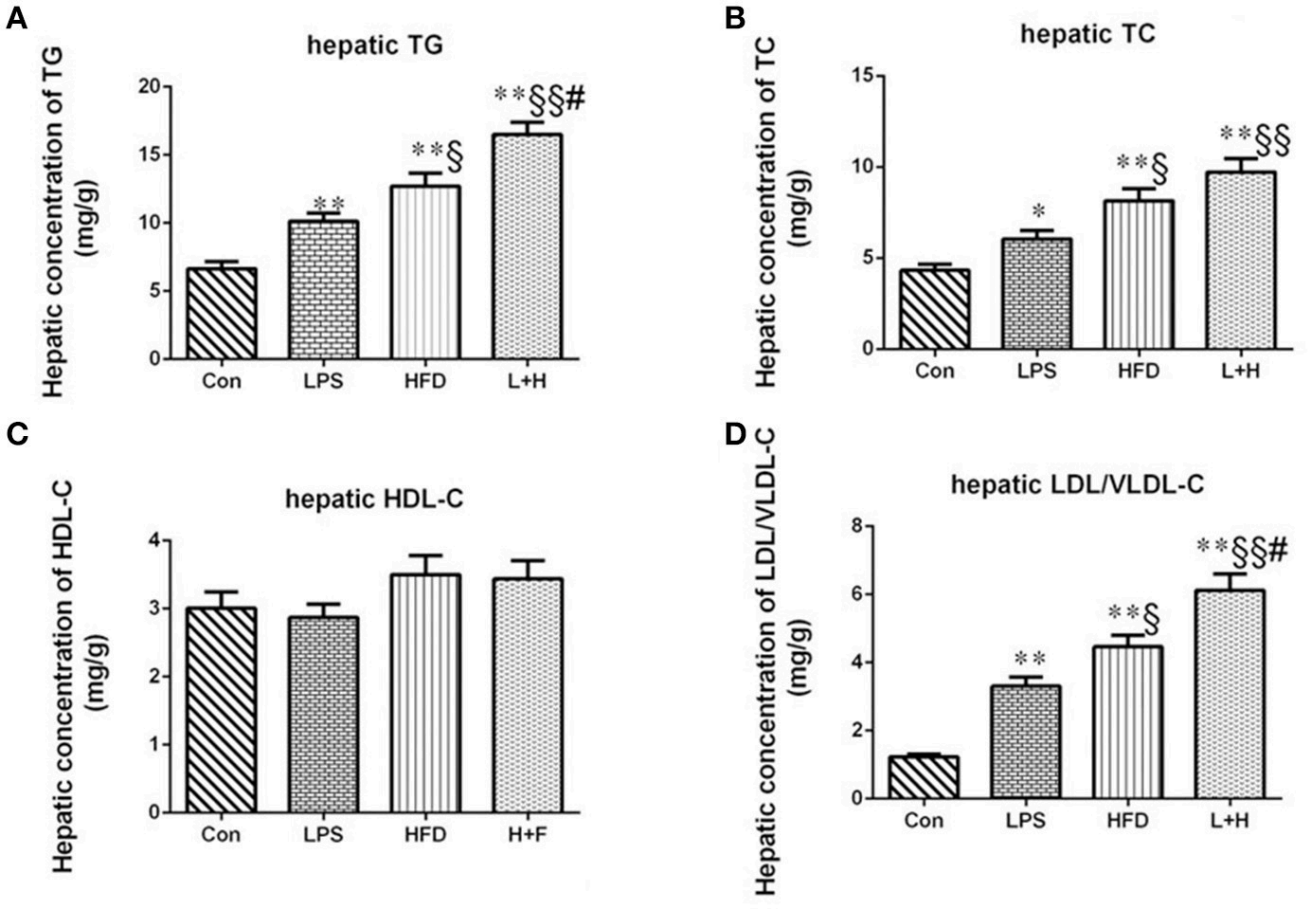
Hepatic contents of triglycerides **(A)**, total cholesterol **(B)**, high-density lipoprotein cholesterol **(C)**, low/very low-density lipoprotein cholesterol **(D)** in 8-week-old offspring rats. The data are presented as the mean ± SEM. *n* = 6 in each group. ^*^P < 0.05, ^**^P < 0.01 vs. control, ^§^*P* < 0.05, ^§§^*P* < 0.01 vs. LPS group, ^#^*P* < 0.05 vs. HFD group (One-way ANOVA). Con group, male offspring rats treated with prenatal saline and postnatal normal diet from 4 to 8 weeks old; LPS group, male offspring rats treated with prenatal LPS and postnatal normal diet from 4 to 8 weeks old; HFD group, male offspring rats treated with prenatal saline and postnatal high fat diet from 4 to 8 weeks old; L+P group, male offspring rats treated with prenatal LPS and postnatal high fat diet from 4 to 8 weeks old.

The assessment of hepatic lipid profiles indicated that prenatal LPS exposure, postnatal HFD feeding and combination treatment all produced remarkable increase in hepatic TG (*P* < 0.01), TC (*P* < 0.01 or 0.05) and LDL-C (*P* < 0.01) in the offspring at 8 weeks of age by comparison of those in control group. Compared with offspring rats from LPS-exposed dams, offspring rats treated with HFD alone or prenatal LPS exposure accompanying 4 weeks of HFD both exerted higher contents of TG (*P* < 0.05 or 0.01), TC (*P* < 0.05 or 0.01) and LDL-C (*P* < 0.05 or 0.01). In addition, prenatal LPS exposure and postnatal HFD feeding synergistically showed significantly increased contents of hepatic TG (*P* < 0.05) and LDL-C (*P* < 0.05) in the offspring compared with the offspring that were solely exposed to postnatal high fat diet. The hepatic HDL-C content, however, did not achieve obvious difference among the four groups (Figure 3).

### Liver morphological damages in offspring rats

**Figure 4:**
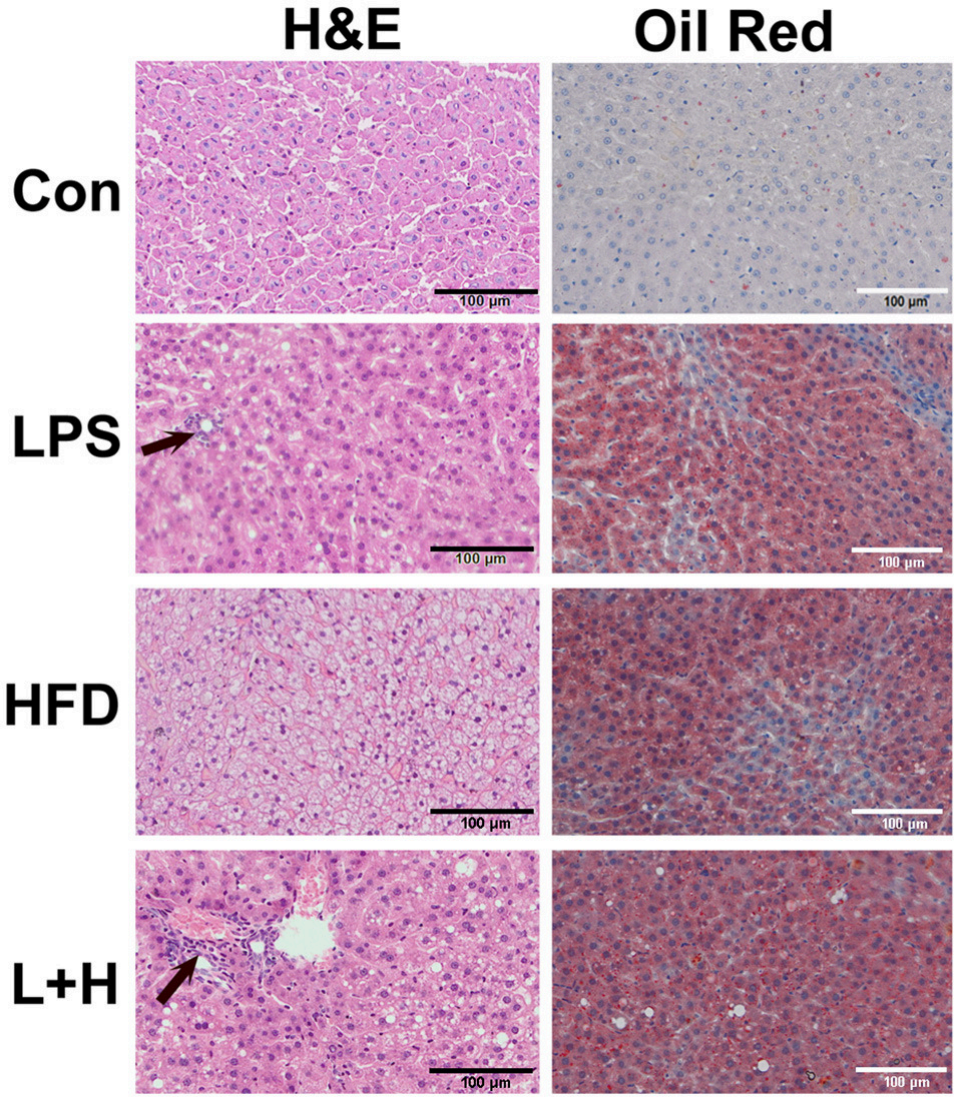
Histopathologic observation of the liver in 8-week-old offspring rats. H&E staining was in left line and oil red staining was in right line. Arrow represented lymphomonocytic perivenular infiltration in liver. Con group, male offspring rats treated with prenatal saline and postnatal normal diet from 4 to 8 weeks old; LPS group, male offspring rats treated with prenatal LPS and postnatal normal diet from 4 to 8 weeks old; HFD group, male offspring rats treated with prenatal saline and postnatal high fat diet from 4 to 8 weeks old; L+P group, male offspring rats treated with prenatal LPS and postnatal high fat diet from 4 to 8 weeks old.

Observation of the liver morphology in the 8-week-old offspring showed normal hepatic architecture without lipid accumulation in the control offspring. Simultaneously, few lipid droplets and marked mixed lymphomonocytic perivenular infiltration in the livers were observed in the prenatal LPS exposure group. Treatment of a high fat diet alone led to a large number of ballooning hepatocytes as well as extensive fat accumulation in the liver. Comparatively, the offspring exposed to prenatal LPS and postnatal high fat diet feeding generated widespread lipid vacuole deposition inside the liver, accompanied with evident lymphomonocytic infiltration (Figure 4). Semi-quantitative analysis indicated that offspring rats from not only LPS-exposed dam but also postnatal treatment with HFD alone achieved a score of 2, suggesting histopathology Kleiner scoring as mild NAFL. Additionally, prenatal LPS exposure following postnatal HFD generated a score of 4, which is compatible with a diagnosis of moderate NAFL (Table 1). These findings demonstrated that exposure to a postweaning HDF likely caused hepatic steatosis and this effect was obviously exaggerated when the offspring were also exposed to LPS in the early developmental period.

**Table 1:** Quantify the histological changes in liver according to the Kleiner scoring system.

	**Steatosis**	**Ballooning**	**Lobular inflammation**	**Activity score**	**Indication**
Con	0	0	0	0	Normal
LPS	0	0	1	1	NAFL
HFD	1	1	0	2	NAFL
L+H	2	1	2	5	NASH

*For each group, the mean score about each feature is given (n = 3)*.

### The dysregulation of gene expression involved in hepatic lipid metabolism and other related pathways in offspring rats

**Figure 5:**
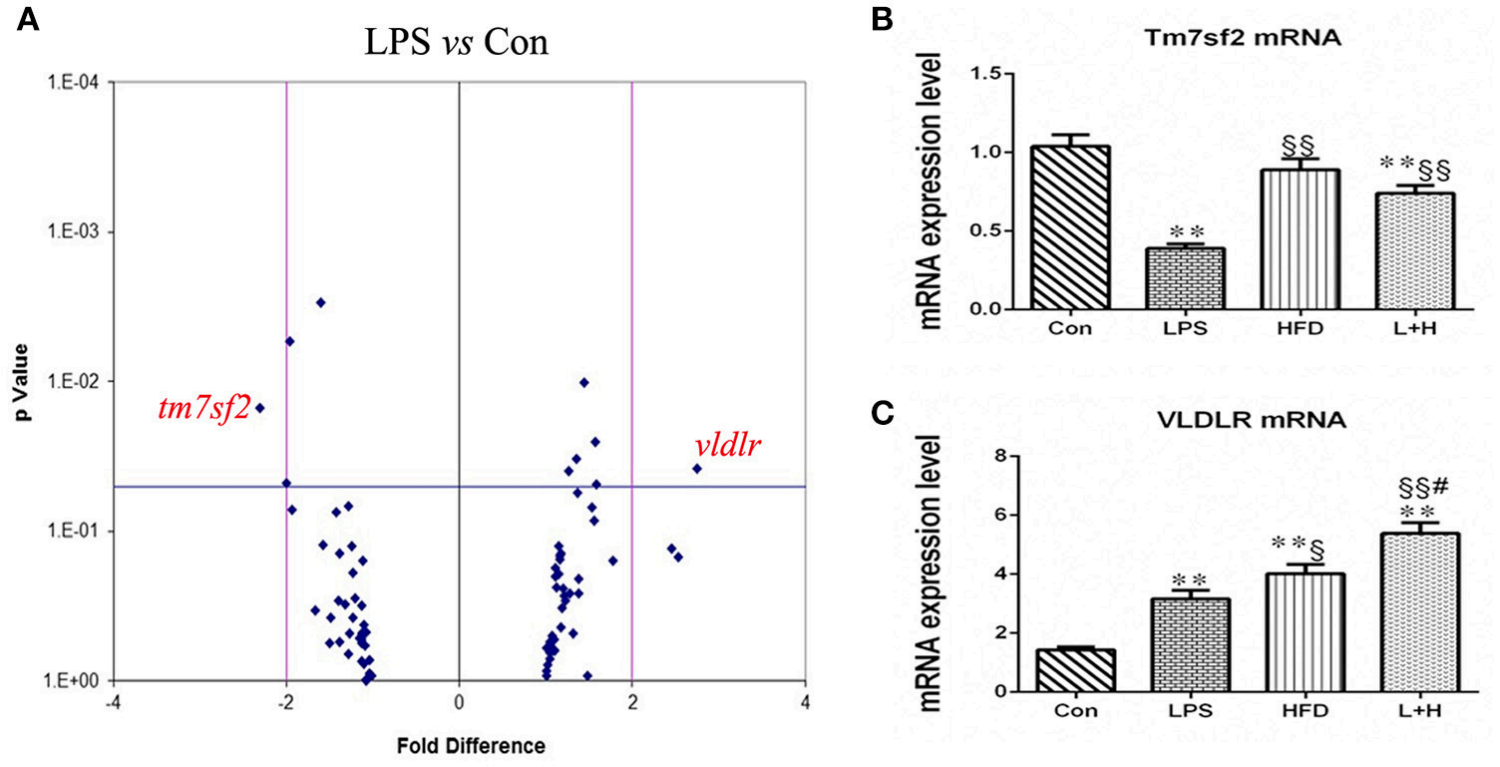
Hepatic gene expression related to lipid metabolism and other related pathways in 8-week-old offspring rats. **(A)** Differentially expressed genes between the control and LPS groups. Differentially expressed genes detected by RT-PCR array. A 2-fold difference and *p* value = 0.05 were marked as vertical and horizontal lines, respectively. *n* = 3 in each group. mRNA expressions of Tm7sf2 **(B)** and VLDLR **(C)** in the livers of offspring rats. The data are presented as the mean ± SEM. *n* = 6 in each group. ^**^P < 0.01 vs. control, ^§^*P* < 0.05, ^§§^*P* < 0.01 vs. LPS group, ^#^*P* < 0.05 vs. HFD group (One-way ANOVA). Tm7sf2, transmembrane 7 superfamily member 2; VLDLR, very low density lipoprotein receptor. Con group, male offspring rats treated with prenatal saline and postnatal normal diet from 4 to 8 weeks old; LPS group, male offspring rats treated with prenatal LPS and postnatal normal diet from 4 to 8 weeks old; HFD group, male offspring rats treated with prenatal saline and postnatal high fat diet from 4 to 8 weeks old; L+H group, male offspring rats treated with prenatal LPS and postnatal high fat diet from 4 to 8 weeks old.

A target-specific qPCR array for genes related to lipoprotein transport and cholesterol metabolism was employed to detect the effect of prenatal LPS exposure on hepatic lipid metabolism. Table 1 showed a summary of the results that were modified with > or = 1.3-fold changes and statistically significant (*P* < 0.05). We found that 4 genes, including cytochrome P450, family 46, subfamily a, polypeptide 1 (CYP46A1), peroxisome proliferator-activated receptor delta (PPAR-δ), protein kinase, AMP-activated, alpha 2 catalytic subunit (PRKAA2) and very low density lipoprotein receptor (VLDLR) were up-regulated, whereas cytochrome P450, family 7, subfamily b, polypeptide 1 (CYP7B1), 24-dehydrocholesterol reductase (DHCR24), transmembrane 7 superfamily member 2 (TM7SF2) and oxidized low density lipoprotein (lectin-like) receptor 1(OLR1) were down-regulated in the offspring rats of LPS-treated dams. Furthermore, the differentially expressed genes were grouped into categories corresponding to LDL receptors (OLR1 and VLDLR), cholesterol catabolism (CYP46A1), cholesterol biosynthesis (DHCR24, PRKAA2, TM7SF2) and other cholesterol metabolism pathways (CYP7B1, PPARD, VLDLR). The expression of the genes related to cholesterol absorption and transport such as ATP-binding cassette, subfamily A member 1 (ABCA1), ATP-binding cassette, subfamily G member 1 (abcg1), and low density lipoprotein receptor (LDLR), however, did not present significant difference between this two groups. Noticeably, the gene expression of TM7SF2 and VLDLR were remarkably altered, showing down-regulation by 2.31-folds and up-regulation by 2.75-folds, respectively, in offspring from LPS exposed dams (Table 2, Figure 5A).

**Table 2:** Differentially expressed genes involved in hepatic lipid metabolism and other related pathways between control and prenatally exposed offspring rats (*n* = 3).

**Symbol**	**Full name**	**Fold**	***P*-value**
Cyp46a1	Cytochrome P450, family 46, subfamily a, polypeptide 1	1.5750	0.025
Cyp7b1	Cytochrome P450, family 7, subfamily b, polypeptide 1	−1.9607	0.005
Dhcr24	24-dehydrocholesterol reductase	−1.5962	0.0029
Olr1	Oxidized low density lipoprotein (lectin-like) receptor 1	−1.9972	0.047
Ppard	Peroxisome proliferator-activated receptor delta	1.3554	0.033
Prkaa2	Protein kinase, AMP-activated, alpha 2 catalytic subunit	1.4393	0.01
Tm7sf2	Transmembrane 7 superfamily member 2	−2.3102	0.015
Vldlr	Very low density lipoprotein receptor	2.7549	0.038

Subsequently, we determined the mRNA expression of VLDLR and TM7SF2 in different treatment offspring rats using quantitative RT-PCR. Compared with the control offspring, the mRNA expression level of Vldlr was significantly higher in the offspring that received a single prenatal LPS exposure (LPS vs. Con, *P* < 0.01), the postnatal high-fat diet (HFD vs. Con, *P* < 0.01) or the L+H combination treatment (L+H vs. Con, *P* < 0.01). Furthermore, increased Vldlr mRNA expression was more obvious in the offspring fed HFD compared with those from LPS-exposed dam (HFD vs. LPS, *P* < 0.05). Prenatal LPS exposure and postnatal HFD feeding synergistically produced significantly increased mRNA expression of VLDLR in the offspring when compared with the offspring that were solely exposed to prenatal LPS (L+H vs. LPS, *P* < 0.01) or postnatal HFD (L+H vs. HFD, *P* < 0.05). The mRNA expression of Tm7sf2, however, was markedly lower in the offspring with prenatal LPS exposure (LPS vs. Con, *P* < 0.01) and in the combination treatment offspring (L+H vs. Con, *P* < 0.01) by comparison of those in control group. Compared with the prenatal LPS exposure offspring, TM7SF2 mRNA expression increased obviously in the combination treatment group (L+H vs. LPS, *P* < 0.01) (Figures 5B,C).

### The mitochondrial membrane potential in offspring rats

**Figure 6:**
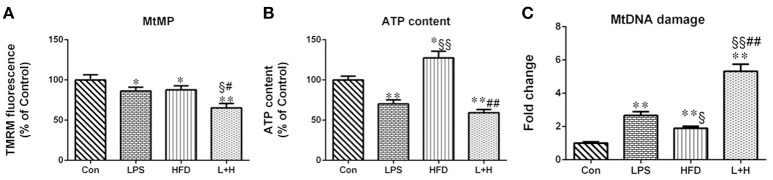
Hepatic mitochondrial membrane potential, **(A)**, ATP content **(B)** and mitochondrial DNA (MtDNA) damage **(C)** in 8-week-old offspring rats. The result about mitochondrial membrane potential and ATP content is expressed as % control, however, MtDNA damage is represented as “fold increase” over the controls in mitochondrial common deletion. The data are presented as the mean ± SEM. *n* = 6 in each group. ^*^P < 0.05, ^**^P < 0.01 vs. control, ^§^*P* < 0.05, ^§§^*P* < 0.01 vs. LPS group, ^#^*P* < 0.05, ^##^*P* < 0.01 vs. HFD group (One-way ANOVA). Con group, male offspring rats treated with prenatal saline and postnatal normal diet from 4 to 8 weeks old; LPS group, male offspring rats treated with prenatal LPS and postnatal normal diet from 4 to 8 weeks old; HFD group, male offspring rats treated with prenatal saline and postnatal high fat diet from 4 to 8 weeks old; L+H group, male offspring rats treated with prenatal LPS and postnatal high fat diet from 4 to 8 weeks old.

The capacity of the mitochondria to generate membrane potential was significantly reduced in hepatic mitochondria from the maternal LPS exposure offspring (LPS vs. con, *P* < 0.05), offspring fed a high-fat diet (HFD vs. Con, *P* < 0.05), as well as the combination treatment offspring (L+H vs. con, *P* < 0.01) compared with the control offspring. In addition, a solely exposure to prenatal LPS or a postnatal high-fat diet did not exert obviously different effects on hepatic mitochondrial membrane potential in offspring. Nevertheless, the offspring from the LPS-exposed dams that received a postnatal high-fat diet showed reliably decreased mitochondrial membrane potential compared with offspring prenatally exposed to LPS (L+H vs. LPS, *P* < 0.01) or control offspring fed a high-fat diet alone (L+H vs. HFD, *P* < 0.05) (Figure 6A).

### ATP content in offspring rats

The hepatic ATP level in prenatal LPS exposure offspring, offspring exposed to maternal LPS and postnatal high-fat feeding was 29.84 and 31.68% less than in the control offspring rats, respectively. Conversely, hepatic ATP levels in the offspring treated with a postnatal HFD alone showed a remarkable increase compared with both the control offspring and the prenatal LPS exposure offspring (HFD vs. LPS, *P* < 0.01). Compared with the offspring with a single exposure to a high-fat diet or prenatal LPS, the combination treatment induced a significant decrease (L+H vs. HFD, *P* < 0.05; L+H vs. LPS, *P* < 0.05) in hepatic ATP levels in the offspring rats (Figure 6B).

### Mitochondrial “common” deletion in offspring rats

MtDNA damage was assayed by identifying mitochondrial “common” deletion. The complete 16-kb mitochondrial genome encodes 37 genes and the 4977-bp MtDNA common deletion, from nucleotide positions 8470 to 13447 bp, spans 5 tRNA genes and 7 genes encoding mitochondrial respiratory chain polypeptides: ATP synthase subunit 6 (Complex V), cytochrome c oxidase (Complex IV), 4 polypeptides of NADH coenzyme Q reductase (Complex I) (ND3, ND4, ND4L, and ND5), and 5 small tRNA (Andrews et al., 1999; Dani et al., 2004). Compared with the control offspring, the rate of common deletion in the liver was increased 2.66, 1.9 and 5.32 times in the prenatal LPS exposure offspring (LPS vs. Con, *P* < 0.01), the high-fat diet fed offspring (HDF vs. Con, *P* < 0.01) and offspring receiving combination treatment (L+H vs. Con, *P* < 0.01), respectively. Additionally, the HFD exposure alone in the offspring produced the lower rate of common deletion compared with the prenatal LPS exposure offspring (HFD vs. LPS, *P* < 0.05). Simultaneously, combined treatment dramatically increased the rate of common deletion compared with the offspring that received a single exposure to prenatal LPS (L+H vs. LPS, *P* < 0.01) or a postnatal high-fat diet (L+H vs. HFD, *P* < 0.01) (Figure 6C).

### The expression of hepatic Complex I, II, III, IV, and V in offspring rats

**Figure 7:**
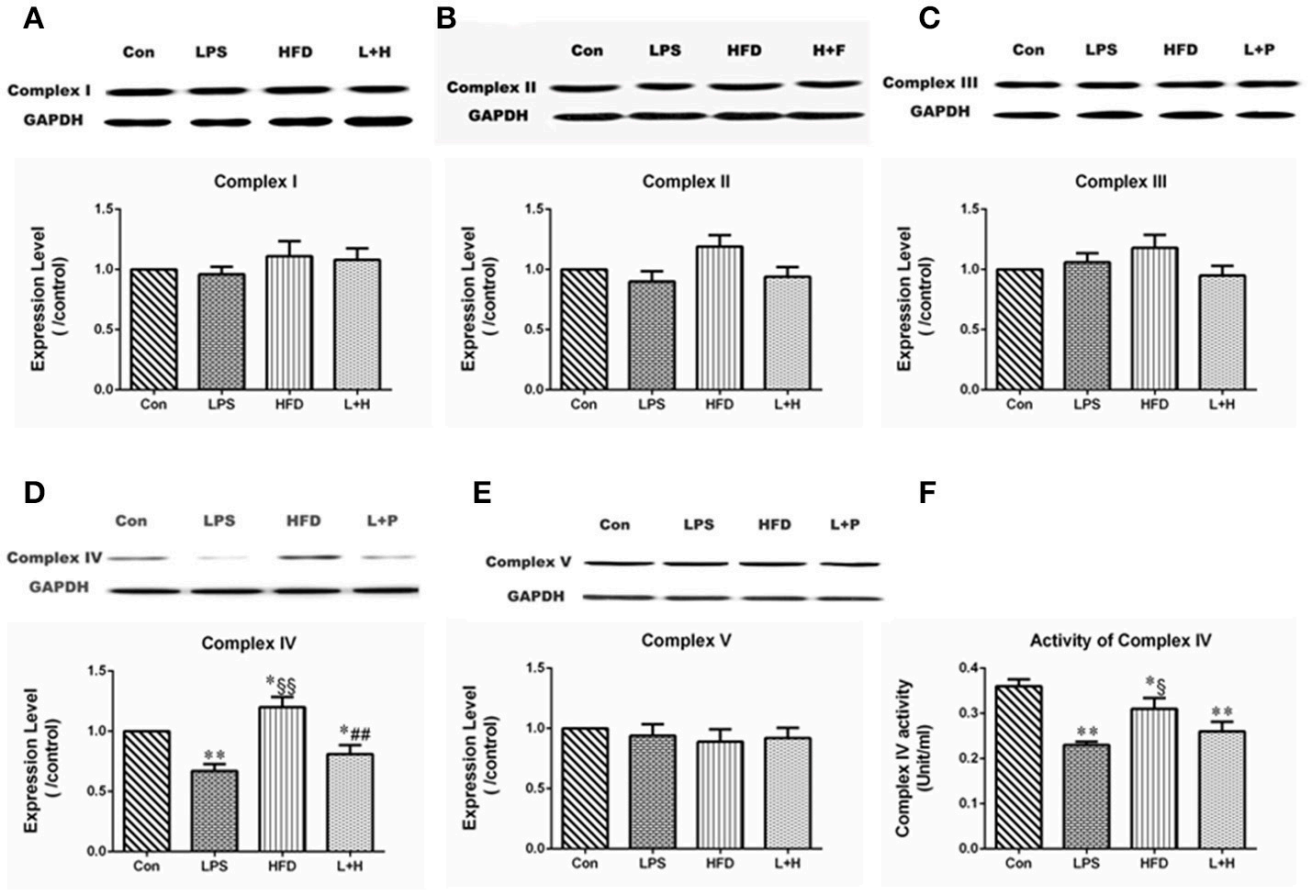
The alterations in mitochondrial respiratory complex proteins in 8-week-old offspring rats. protein expression of mitochondrial respiratory complex I **(A)**, II **(B)**, III **(C)**, IV **(D)**, and V **(E)**. The activity of mitochondrial respiratory complex protein IV **(F)**. The data are presented as the mean ± SEM. *n* = 6 in each group. ^*^P < 0.05, ^**^P < 0.01 vs. control, ^§^*P* < 0.05, ^§§^*P* < 0.01 vs. LPS group, ^##^*P* < 0.01 vs. HFD group (One-way ANOVA). Con group, male offspring rats treated with prenatal saline and postnatal normal diet from 4 to 8 weeks old; LPS group, male offspring rats treated with prenatal LPS and postnatal normal diet from 4 to 8 weeks old; HFD group, male offspring rats treated with prenatal saline and postnatal high fat diet from 4 to 8 weeks old; L+H group, male offspring rats treated with prenatal LPS and postnatal high fat diet from 4 to 8 weeks old.

To further evaluate whether MtDNA damage correlated with changes in the expression or activity of the mitochondrial respiratory complexes, we detected the protein expression and activity of respiratory complex subunit in the liver. Compared with the control offspring, maternal LPS exposure caused obviously lower expression of Complex IV (LPS vs. Con, *P* < 0.01) in 8-week-old offspring. Although the expression of Complex II, III, and IV in offspring treated with HFD alone didn't show significant difference by comparison of those in offspring from control or LPS group, the expression of Complex IV increased significantly compared with the control offspring (HFD vs. Con, *P* < 0.05) and offspring from the LPS-exposed dams (HFD vs. LPS, *P* < 0.01), respectively. Additionally, combination treatment led to markedly decreased expression of Complex IV compared with the control (L+H vs. Con, *P* < 0.01) and single high-fat feeding offspring (L+H vs. HFD, *P* < 0.05) (Figures 7A-E).

### Complex IV activity in offspring rats

The enzymatic activity of Complex IV in the liver was dramatically lower in offspring exposed to prenatal LPS (LPS vs. Con, *P* < 0.01), postnatal high-fat diet (HFD vs. Con, *P* < 0.05) and combination treatment (L+H vs. Con, *P* < 0.01) than in the control offspring. In addition, postnatal high-fat feeding exerted obviously increased enzymatic activity of Complex IV compared to the results in offspring from the LPS-exposed dams (HFD vs. LPS, *P* < 0.05) (Figure 7F).

## Discussion

In this study, the major findings were that maternal exposure to LPS during pregnancy led to disorders of lipid profiles in 8-week-old male offspring rats, characterized by remarkably elevated levels of TC, TG, and LDL-C, even under the standard chow feeding. It has been suggested that maternal LPS treatment during pregnancy accelerated the development of adult cardiovascular related diseases, including hypertension, obesity, atherosclerosis, cardiac hypertrophy (Wei et al., 2007, 2013; Zhao et al., 2014). To the best of our knowledge, these current data provided, for the first time, direct experimental evidence supporting the notion that prenatal exposure to LPS evokes a lipid profile shift that could alter the offspring's risk of CVD later in life. Diet, as related to the intake of saturated fat and cholesterol, has been shown in a number of epidemiologic studies to be an important risk factor in the development of dyslipidemia, atherosclerosis and other cardiovascular and metabolic diseases (LaRosa et al., 1990; American Heart Association Nutrition Committee et al., 2006). Our results demonstrated the more prominent dysfunction of blood lipid profiles and excessive weight gain in offspring exposed to prenatal LPS following a high fat diet from 4- to 8-weeks of age, underlining the inherent vulnerability induced by the poor intrauterine environment that should become apparent in the face of risk factors such as excessive dietary fat in adulthood. It is notable that our present study was confined to male offspring rats, as females would have required the assessment of estrus due to the impact of estrogen on lipid metabolism (Faulds et al., 2012; Kolovou et al., 2014).

The liver plays a critical physiological role in the regulation of lipoproteins, cholesterol, and TG. In our study, however, maternal LPS administration produced adverse liver structural and functional changes, accompanying with hepatic TG accumulation in offspring rats at the age of 8 weeks old. It is widely accepted that a poor prenatal environment can modify postnatal physiology through a process known as “programming,” including structural and cellular changes in fetus to ensure short-term survival but that may also result in impaired structure and function in the later life. Maternal LPS administration at conception has been shown to elevate levels of TNF-α and IL-6 in maternal serum and placenta after 2 h of the last LPS administration, while only IL-6 mRNA expression in the fetus was obviously increased after 48 h of LPS injection. In comparison to TNF-α and IL-6 rapid and robust increases in maternal serum and placenta, the delayed elevation of IL-6 in fetus uncovered that the alterations in embryonic development might be caused indirectly by maternal derived pro-inflammatory cytokines exposure as well as directly from *in utero* LPS (Deng et al., 2016). We previously have displayed that maternal LPS exposure during pregnancy exerted profound programming effects on the heart, coronary blood vessels, kidneys, and adipose tissue in offspring rats (Gao et al., 2014; Zhao et al., 2014; Chen et al., 2015; Zhang et al., 2016). In view of programming, which involves adaptive changes in gene expression patterns that occur in response to a stress, we introduced a target-specific qPCR array to detect the gene expression involved in hepatic lipid metabolism and related pathways in the offspring rats. In the present study, prenatal exposure to LPS triggered remarkable alterations in the gene expression of VLDLR, showing up-regulation by 2.75-folds. VLDLR is a member of the LDL receptor superfamily. In peripheral tissues, VLDLR binds apolipoprotein E-triglyceride-rich lipoproteins (TRLs) and mediates their uptake (Takahashi et al., 1995). Alternatively, long-term stable overexpression of VLDLR in the liver of LDLR-deficient mice increased liver TG content, although it improved plasma lipid profiles and aortic atherosclerosis (Oka et al., 2001). While HepG2 cells stably overexpressing VLDLR manifested increased intracellular triglyceride accumulation, VLDLR-deficient mice were protected against fatty liver (Wang et al., 2014). In cardiomyocytes, hypoxia/ischemia-induced accumulation of lipids was dependent on the expression of the VLDLR (Perman et al., 2011). Despite the adaptability of TM7SF2 gene to the needs of cholesterol biosynthesis seemed well established (Bennati et al., 2008), our data shown maternal LPS administration evoked remarkable down-regulation (2.31-folds) in offspring rats. Recent study has revealed overexpression of hTM7SF2 protein could reverse the inflammatory phenotype (Bellezza et al., 2013). Thus absence of TM7SF2 gene presented the local inflammatory phenotype, keeping line with the higher mRNA expression of pro-inflammatory cytokines, such as IL-1β, IL-18, and TNF-α in liver from prenatal LPS challenged offspring rats (data not shown). In addition, other genes in the offspring from the LPS exposed dams, including CYP46A1, PPARD, PRKAA2, CYP7B1, DHCR24, and OLR1, which are responsible for different links during lipid metabolism, all appeared to have moderately altered expression levels. Collectively, perturbation of hepatic genes involved in lipid metabolism revealed a metabolic shift favoring enforced production of lipid in prenatal LPS treatment offspring rats and suggested a predispose to high fat diet induced enforced dyslipidemia. Furthermore, the absence of TM7SF2 gene, which also implied that hepatic inflammation should not be ruled out the contribution to lipid abnormalities. However, in our case, the evidence of a clear relationship still requires further investigations.

Mitochondria govern a central hub in hepatic lipid metabolism and also are affected by upstream signaling pathways involved in hepatic metabolism. A mitochondrion contains inner and outer membranes composed of phospholipid bilayers and proteins. The electron transport chain (ETC) is embedded in the inner mitochondrial membrane that comprises four enzymatic series of complexes (Complexes I–IV) together with ATP synthase, which synergistically work in concert as a biological machine. Consequently, the loss of any complex may be at least partly responsible for impaired ATP biogenesis. The 4977-bp “common” deletion, although found in low abundance, is frequently observed in the pathogenesis of chronic liver disorders, atherosclerosis and aging-related diseases, indicating mitochondrial damage (Botto et al., 2001; Pavicic and Richard, 2009; Guo et al., 2017). In this study, we found evidence of hepatic mitochondrial dysfunction and mitochondrial DNA (MtDNA) damage in offspring from dam received LPS stimulation during pregnancy, as confirmed by the loss of ATP content, diminished membrane potential, decline in protein expression and activity of mitochondrial respiratory complexes, as well as increased level of 4977-bp MtDNA common deletion, suggesting a precedent for “priming” of the mitochondria, which persisted into adulthood. Regarding lipid dysmetabolism, different mechanisms of mitochondrial dysfunction have been described thus far, including depletion of mtDNA, decreased activity of respiratory chain complexes, and impaired mitochondrial β-oxidation. For example, the lowered ETC activity and expression is likely to compel intensive generation of reactive oxygen species, which have been shown to initiate lipid peroxidation and trigger the release of inflammatory cytokines contributing to the development of dyslipidemia (Begriche et al., 2011). Aluminum should disturb ATP production and mitochondrial function thereby diverting incoming metabolites toward VLDL production (Mailloux et al., 2007). Of note, uteroplacental insufficiency led to hepatic lipid accumulation in offspring rats due to the alterations in the expression of hepatic fatty acid-metabolizing enzymes (Lane et al., 2001). In this context, impaired mitochondrial function may be partially responsible for hepatic lipid imbalance thereby improving serum dyslipidemia in offspring rats from maternal LPS administration.

In this study, notably, mitochondrial dysfunction in the offspring fed a high fat diet alone exhibited the different patterns, including remarkably higher ATP level, an increased tendency toward complex expression and enforced Complex IV expression. However, a sharp reduction in the activity of Complex IV was found, suggesting that the hepatic mitochondria experienced an adaptive response that likely tried to preserve ATP synthesis and mitochondrial balance. Subsequently, the mitochondrial flexibility was lost when the high fat diet lasted 12 weeks, and it was accompanied by augmented lipid dysmetabolism (data not shown). These alterations were consistent with the reports of increased mitochondrial function in obese humans and mice with non-alcoholic steatohepatitis, which was subsequently abolished in steatohepatitis (Buchner et al., 2011; Koliaki et al., 2015). On the another hand, offspring from dams received LPS and postweaning high fat diet feeding exhibited a further worsening mitochondrial dysfunction accompanied by the more exacerbated form of hepatic lipid accumulation and serum lipid profile disorder, underscoring pre-existed mitochondrial disorders contributed to an increased susceptibility to postnatal high fat diet.

It should be mentioned that, being inconsistent with our data, Hao et al. found there was no difference in body weight and blood lipid levels between offspring rats from control and LPS-treated dams. Additionally, Hao et al. also mentioned high fat diet feeding in adulthood couldn't produce enforced form in body weight gain and dyslipidemia in the prenatal HFD and LPS synergistically exposed offspring rats (Hao et al., 2014). We have showed that prenatal LPS (0.79 mg/kg) led to local inflammation, including adipose tissues (Gao et al., 2014) and liver (data not shown), which has been well documented the contribution to obesity and lipid dysmotabolism (Dandona et al., 2005; Jung and Choi, 2014). Of note, 0.40 mg/kg of LPS other than 0.79 mg/kg was administrated in pregnancy rats in Hao's study, we speculated that the dosage difference should likely contribute to the different results. According to Hao et al' statement that blunt sensitivity to postweaning high fat diet could be the result of interaction between prenatal LPS treatment and high fat diet. However, we employed model of maternal exposure to LPS alone to assess the susceptibility in offspring. Therefore, predictive adaptive response occurred in Hao's model could not be detected in our study. Overall, we should pay attention to the influence of different dosage of LPS, even synergism of different stress during early life on adult diseases.

## Limitation

The present study laid emphasis on the impacts of maternal LPS exposure during pregnancy on lipid profiles in male offspring. We were unable to define the underlying relationship between the disturbed gene network involved in hepatic lipid metabolism and impaired mitochondrial function. However, it has been documented that the connection between cholesterol metabolism and inflammation was exemplified by the TM7SF2 gene, and defects in cholesterol biosynthesis might in turn disrupt the finely tuned regulation of the transcriptional factors related to the inflammatory response (Gatticchi et al., 2015). There is increasing evidence that mitochondrion locate at the convergent point maintaining inflammation and lipid metabolism (Blas-García et al., 2016; Thoudam et al., 2016). Thus, down-regulated gene expression of TM7SF2 served to mitochondrial dysfunction and lipid profile abnormalities, which should need to be extensively studied.

## Conclusions

This study highlights that exposure to LPS during early periods of development could lead to lipid profile disorder in male offspring rats, in which programming expression of genes related to lipid metabolism and mitochondrial function was involved in. Specifically, as the offspring rats encounter a high fat diet in adult environments, these developmentally reprogramming phenotypes are exaggerated, resulting in a florid dyslipidemia. Overall, this finding may shed light on a novel risk factor for dyslipidemia. Therapeutic strategies to inflammation regulation in early life may therefore provide a new approach to prevent CVDs and metabolic diseases in adulthood.

## Author contributions

YL and HZ: designed and performed the experiments, drafted and revised the manuscript, and prepared the final version of the manuscript; SY, YW, and JL: performed the experiments; YL: analyzed and interpreted the data. All authors read and approved the version submitted for publication.

### Conflict of interest statement

The authors declare that the research was conducted in the absence of any commercial or financial relationships that could be construed as a potential conflict of interest.
