# Supplementary material for: Prenatal Lipopolysaccharide Exposure Promotes Dyslipidemia in the Male Offspring Rats
Source: Front Physiol. 2018 May 16;9:542. doi: 10.3389/fphys.2018.00542 (PMC5964359; doi:10.3389/fphys.2018.00542)
Supplement: Supplementary file 1 [file Table_1.pdf]

SUPPLE. TABLE 1. Major dietary compositions by calories in percent

	Control Diet	High Fat Diet
Protein	**20**	**20**
Casein	19.7	19.7
Carbohydrate	**70**	**20**
Corn starch	31	0
Sucrose	34.5	6.8
Maltodextrin	3.5	12.3
Fat	**10**	**60**
Soybean oil	5.5	5.5
Lard	4.5	54.35

Numbers in bold represent total percent kilocalories as protein, carbohydrate, and fat. The rest of numbers demonstrate the individual catabolism of protein, carbohydrate, and fat by calories in percent.
